# Do Commercially Available Insoles Meaningfully Change Reported Comfort and Biomechanics During One Day of Wear?

**DOI:** 10.1002/jfa2.70163

**Published:** 2026-05-14

**Authors:** Max Lewin, Richard Jones, Carina Price

**Affiliations:** ^1^ Centre for Human Movement and Rehabilitation School of Health and Society University of Salford Salford Manchester UK

**Keywords:** biomechanics, comfort, insole

## Abstract

**Introduction:**

Identifying meaningful changes in comfort and any underpinning biomechanical changes is crucial to understanding differences in perceptions of footwear comfort. Research rarely considers changes in comfort, and rarely reports how meaningful these are. This study aims to utilise a portable biomechanics measurement tool to establish whether commercially available insoles make meaningful changes in comfort and identify any concurrent changes in biomechanics compared with a control whilst worn during a users' normal day.

**Materials and Methods:**

Thirty participants wore five insole conditions (participants' own shoe, and four commercially available insoles) for a typical day whilst the Runscribe inertial measurement unit (IMU) captured: Ground reaction force (GRF), GRF rate, pronation excursion, pronation velocity, impact shock, braking shock, and total shock. Comfort was measured using a 100 mm visual analogue scale. A pre‐defined ± 8.28 mm minimal clinical important difference (MCID) for comfort was used as the threshold of meaningful change to group individuals into comfort classifications: ‘more’ (47 participants) and ‘less’ (36 participants). MCID was also defined for biomechanics variables from the RunScribe. Insole conditions were compared between classifications to identify significant and meaningful changes in biomechanics associated with meaningful changes in comfort from the control condition. Paired sample t‐tests compared biomechanical parameters from the RunScribe between comfort classifications.

**Results:**

Similar numbers of individuals reported ‘more’ and ‘less’ comfort across conditions. There were only significant reductions in braking shock when individuals reported meaningfully ‘less comfort’. The primary result from meaningful change thresholds for biomechanics was meaningful increases in impact shock when comfort was both ‘more’ and ‘less’.

**Discussion:**

The measurement approach enabled identification of comfort changes, and underpinning biomechanical differences during one day of wear of insoles. Impact shock was the main contributor to comfort perception, consistent with previous literature. Highlighting impact shock as a potential variable for assessment of interventions in pathological study.

## 1 Introduction

Footwear comfort is crucial to both wearers and manufacturers to access and produce comfortable footwear. Comfort influences adherence in clinical settings [1], and is linked to likelihood of purchase [2]. In active populations, improved comfort is associated with reduction of injury risk [3], and research within running suggesting individuals should select footwear based upon comfort rather than biomechanical requirements [4]. Comfort ultimately underpins the initial decision to wear, and adherence to footwear‐based interventions [5, 6, 7]. Therefore, it is essential to understand the factors contributing to comfort perceptions of wearers.

The relationships between lower limb biomechanics and footwear and insole comfort have been explored [8, 9]. The challenge surrounding insoles is the ability to create meaningful changes in biomechanical gait parameters to consequently drive meaningful changes in comfort. Many studies assessing the use of commercially available insoles report limited differences in either biomechanics or comfort between insole conditions [10, 11]. Limited design scope surrounding dimensions, material placement, and properties, reduce potential for insoles to produce large changes in lower limb biomechanics during walking compared with footwear. Previous research has consistently identified negative correlations between peak pressure and comfort across the foot [12, 13], indicating pressure reduction being key to improving perceived comfort. Further relationships show reductions in kinetic variables, specifically peak vertical ground reaction forces and loading rates, to improve footwear comfort [14, 15]. Increased accelerations measured at the tibia are also linked to reduced footwear comfort and ‘liking’ [14, 16]. Overall, insoles have been shown to improve comfort when used in a range of different shoes [8], and are often utilised for self‐management of foot care for addressing foot pain or deficits in footwear comfort. Improvements in comfort and accessibility highlight the potential use as a clinical intervention to reduce injury, and improve adherence to footwear‐based interventions.

Alongside relatively small changes in biomechanical variables, changes in comfort with insole interventions are sometimes also small in magnitude [17, 18, 19]. When using a visual analogue scale (VAS) to record comfort, the MCID has been described as an important indicator of meaningful change in comfort. The MCID for comfort of running shoes on a 100 mm VAS anchored by ‘not comfortable at all’ and ‘most comfort imaginable’ has been defined as 8.28 mm using the standard error of measurement (SEM) approach [20]. Differences of comfort greater than 8.28 mm therefore indicate a meaningful change. In a practical sense this identifies whether changes to a footwear or insole design make a large enough change in comfort to be meaningful to a wearer rather than defining differences through traditional statistical testing [21, 22, 23] which might not relate to a noticeable change in comfort sensation for the wearer. When paired with biomechanical data collection, this would enable identification of how lower limb biomechanics changes when individuals report comfort changes, that is, if something is ‘more’ or ‘less’ comfortable.

Footwear comfort measurement is often completed alongside biomechanical testing in a laboratory, whereby wearers complete a small number of steps within a controlled environment [24, 25, 26]. This ensures replication of activity, which is not necessarily representative of the environment the insole product will eventually be used. Ensuring external validity of testing environments for users of orthotics is essential to interpret outcomes to assess and tailor treatments and interventions utilised by clinical groups. This study provides an initial exploration into the perceptions of footwear comfort that occur resulting from one day of usage and biomechanical assessment of insole performance outside of a laboratory.

### 1.1 Aims and Hypothesis

The aims of this study are to determine if commercially available insoles make meaningful changes to reported comfort in asymptomatic wearers over a one‐day period. Then to subsequently identify the concurrent significant or meaningful biomechanical changes when compared against a control condition of no insole.

It is hypothesised that individuals reporting meaningful increases in comfort from control to insole, will experience decreases in kinetics (GRF and GRF rate), kinematics (pronation excursion and pronation velocity), and accelerations (impact, braking, and total shock) as measured by the RunScribe Inertial Measurement Unit (IMU). This is based upon a prior systematic review of comfort identifying reductions in all of the above variables alongside improved footwear comfort.

## 2 Materials and Methods

### 2.1 Participants

Thirty participants (male = 15, female = 15; Age = 31.9 ± 10.8 years, Height = 173 ± 9 cm, Body Mass = 72.7 ± 12.5 kg) participated in the study following recruitment using convenience sampling. The selected participant number reflected participant numbers in previous research assessing biomechanical differences between insoles [27, 28]. Favourable opinion was given by the University of Salford School of Health and Society Ethics Committee (#6395) and all participants provided written informed consent prior to participation. Participants were excluded if they were > 65 years of age, had current injury to the lower limbs or lower back, or had history of lower limb or lower back surgery within the previous year.

### 2.2 Lower Limb Biomechanics

The RunScribe IMU (Scribe Labs, Moss Beach, California, USA) is a commercially available IMU used for the measurement of biomechanical parameters pertinent to the performance of insole products. This included: GRF, GRF rate, pronation excursion, pronation velocity, impact shock, braking shock, and total shock (Table 1). Previous research has quantified the validity and sensitivity of the RunScribe IMU for the variables within this study [29, 30]. Repeatability was explored using 4 days of repeated measurements with ten participants, and assessed using Intraclass Correlation Coefficient (ICC) (Supporting Information S1).

**TABLE 1:** Definition of variables measured by the RunScribe IMU.

Variable	Definition	Abbreviation	MCID
Ground reaction force (xBW)	Peak vertical GRF	GRF	0.05
GRF rate (N/Kg/s)	Mean vertical force during stance	GRFrate	0.96
Impact shock (g)	Peak positive vertical acceleration	IS	0.08
Braking shock (g)	Peak negative horizontal acceleration	BS	0.09
Total shock (g)	Vector combination of impact and braking shock	TS	0.10
Pronation excursion (°)	Amount of rotation from initial foot contact to maximum pronation	PE	0.71
Pronation velocity (°/sec)	Maximum velocity of pronation between initial foot contact and maximum pronation	PV	11.70

**FIGURE 1:**
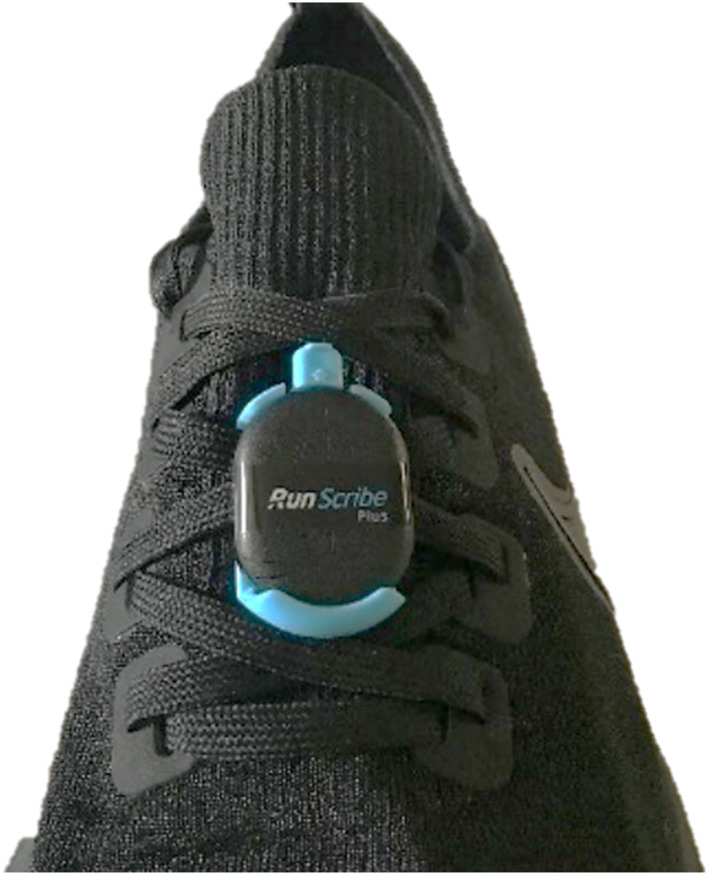
Attachment of the RunScribe IMU to the laces of participant footwear.

RunScribe units were affixed to the laces of the participants’ footwear (Figure 1) and calibrated as per manufacturer instructions. RunScribe was used for the gait measurement throughout the participants’ normal daily activity to quantify biomechanical gait parameters. Participants were instructed to complete the repeated days of measurements on days where they would complete the same activities, and wear the same shoe to house the insole(s).

### 2.3 Insole Conditions

Participants were instructed to wear their own shoes that had removable insoles to accommodate the test conditions or shoes that could accommodate insole conditions without compromising footwear fit. This shoe with the existing insole was defined as the control condition, in line with recommendations from previous research [31]. Each participant then wore four commercially available insole conditions (Table 2), produced by Scholl, Insole conditions (A, B, C, D) were chosen due to their difference in features related to shape, thickness, material type, material properties (defined in Table 2), and assumed influence on biomechanics and perceptions of comfort. Insole A was a multi‐material (memory foam top layer, gel bottom layer, liquid heel pod) insole with a small amount of arch shape. Insole B was constructed the same as insole A however was thicker. Insole C was a thin gel material insole with no arch shape, and insole D was made from foam with a small amount of arch contouring.

**TABLE 2:** Details of insole conditions included within the study.

Insole conditions	Image	Features
Control	NA	Participants own shoe with existing insole
Insole A	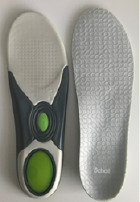	Gel bottom layer, memory foam top layer, liquid heel pod, arch support
Insole B	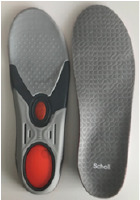	Same composition as insole A, 25% thicker memory foam layer
Insole C	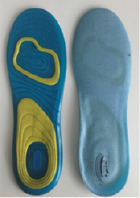	Full gel with no arch shape
Insole D	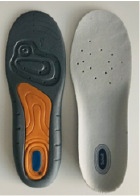	Foam with gel insert from midfoot to heel, minimal arch shape

For the insole conditions, participants wore their own footwear with the existing insole removed and replaced with the test condition. Participants wore each insole condition bilaterally in a randomised order for a full day of wear during which the RunScribe measured gait parameters. Participants were encouraged to replicate activity across days but this differed for each participant, number of steps taken was measured in each of the insole conditions using the RunScribe IMU (Mean ± SD): Control (4058 ± 2404), Insole A (4008 ± 2228), Insole B (4058 ± 3222), Insole C (3962 ± 2349), and Insole D (4556 ± 2873).

Biomechanical comparison of the insole conditions (Table 3) was completed for the whole group (*n* = 30). Following checks for normality and outliers, a Friedman test was completed with a post‐hoc Wilcoxon test with a Bonferroni correction to assess differences between insoles conditions.

**TABLE 3:** Biomechanical comparison of insole conditions for the full study cohort (*n* = 30).

	GRF (xBW)	GRF rate (N/Kg/s)	Total shock (g)	Impact shock (g)	Braking shock (g)	Pronation excursion (°)	Pronation velocity (°/sec)	Comfort
Control	1.19 (0.13)	14.84 (2.03)	3.75 (1.09)	2.20 (0.88)	2.90 (0.77)^*^	8.94 (5.77)	240.51 (11.88)	65.60 (16.50)
Insole A	1.17 (0.11)	14.43 (1.77)	3.56 (1.37)	2.30 (0.96)	2.62 (0.83)	8.68 (4.94)	235.65 (60.64)	67.00 (22.75)
Insole B	1.17 (0.13)	14.42 (1.69)	3.53 (1.32)	2.30 (0.97)	2.56 (0.87)^*^	9.03 (4.73)	226.58 (70.87)^*^ ^a,b^	68.50 (26.75)
Insole C	1.20 (0.09)	14.95 (1.27)	3.63 (1.38)	2.36 (1.13)	2.68 (0.86)	9.97 (6.08)	245.17 (78.77)^*^ ^a^	65.50 (43.25)
Insole D	1.18 (0.07)	14.96 (1.20)	3.62 (1.23)	2.23 (0.81)	2.66 (0.64)	9.65 (4.41)	246.09 (73.96)^*^ ^b^	72.00 (16.00)
Friedman *p*‐value	0.026	0.102	0.141	0.935	0.012	0.141	0.002	0.230

*Note:* Friedman test significant at *p* ≤ 0.05.

^*^ Indicates significant Wilcoxon post‐hoc test (Bonferroni correction *p* ≤ 0.005).

### 2.4 Visual Analogue Scale

A 100 mm VAS was used to measure comfort of each insole condition. VAS is the most frequently used measurement method for comfort of footwear/insole conditions [9]. The left anchor was ‘least comfort imaginable’ with the right anchor being ‘most comfort imaginable’, participants were instructed to mark along the scale to report a comfort score using a touchscreen device. This was completed each day immediately following the wear period where the RunScribe data were collected.

### 2.5 Data Processing

#### 2.5.1 RunScribe IMU

Data from the RunScribe were processed using the internal algorithm, which processes data to give a maximum value for each variable (Table 1) per‐step during the collected data period for both left and right feet. Per‐step data were averaged to give a value for each day, and left and right feet were averaged to give a single value for the data collection period.

#### 2.5.2 VAS

For comfort score, the distance of the mark from the left side of the VASwas measured in millimetres and recorded, where the minimum was 0 and maximum 100.

#### 2.5.3 Comfort Classification Grouping

For each of the test insoles (A–D), participants were grouped into a comfort classification based upon the magnitude of difference in the comfort scores (Control *minus* test condition), using the pre‐defined ‘data‐derived’ MCID for overall comfort using the 100 mm VAS (8.28) [20]. Comfort classifications were assigned as follows: ‘more comfort’ (≥ 8.28) and ‘less comfort’ (≤ 8.28), differences which did not meet these magnitudes were defined as not meaningfully different and are not presented. Comfort classification allowed comparison of the biomechanical measures from the control to the test insoles when individuals reported meaningful changes in comfort. This was completed in all test conditions to identify consistency of relationships across conditions. This meant that some individuals had a comfort classification of ‘more’ in one test condition and ‘less’ in others depending on the comfort scores in each of the insoles.

### 2.6 Biomechanical Comparison

Control conditions were compared with each insole condition within comfort classification groups. Two different comparisons were made independently between the biomechanical data:

1. Meaningful Difference: MCID threshold to identify meaningful difference

The MCID for the lower limb biomechanics variables measured by the RunScribe were determined using the same SEM approach as used in [20]. The MCID was derived from standalone data and presented in Table 3. The repeatability assessment (Supporting Information S1) was completed on 10 individuals collecting data over 4 days, whereby individuals were requested to complete the same daily living activities on each day whilst wearing the same footwear. Biomechanics data were collected for each step over the day, these data were then averaged to give a single daily average for each of the 4 days of data collection.

A [3, 4] intraclass correlation coefficient (ICC [3, 4]) with average measures and absolute agreement was completed using SPSS statistics 28 (IBM, Armonk, New York, USA) to provide a measure of reliability for the biomechanics variables as measured by the RunScribe. Then the standard deviation (SD) was then calculated from the grouped data of all the insole conditions. The SEM was then calculated to provide the MCID.

2. Statistical Difference: t‐tests to identify statistical differences

Paired comparisons were completed to assess differences in lower limb biomechanics between the control (shoe only) and test insoles for each of the comfort classification groups. Data were assessed for normality using the Shapiro‐Wilk test, and outliers, defining the data as parametric. Four *t*‐tests were undertaken for each of the seven variables for each of the two comfort classifications in SPSS statistics 27 (IBM, Armonk, New York, USA). Bonferroni correction was used to correct for multiple (four) comparisons made within each comfort classification, meaning significance was set to *p* ≤ 0.0125.

## 3 Results

### 3.1 Comfort Classification

Comfort classifications were evenly spread in insole A and insole C (Table 4). A larger proportion of individuals reported meaningfully ‘more comfort’ in insole B and insole D than ‘less comfort’ (Table 4).

**TABLE 4:** Participants assigned to each comfort classification grouping for each insole condition.

Insole	Insole more comfortable (*n* (%))	Insole less comfortable (*n* (%))
A	9 (30%)	10 (33%)
B	15 (50%)	8 (27%)
C	9 (30%)	11 (37%)
D	14 (47%)	7 (23%)

### 3.2 Lower Limb Biomechanics

Comparisons of biomechanical outcomes in each insole condition to the control condition for each comfort classification is presented in Table 5. Significant differences between the control and insole conditions within the comfort classifications were limited. Meaningful differences in biomechanics were more common.

**TABLE 5:** Changes in biomechanical gait parameters for each comfort classification.

Comfort classification	Conditions being compared	GRF (xBW)	GRF rate (N/Kg/s)	Total shock (g)	Impact shock (g)	Braking shock (g)	Pronation excursion (°)	Pronation velocity (°/sec)
Insole more comfortable	Control – Insole A	1.22 (0.12) – 1.18 (0.09)	15.26 (1.25) – 14.64 (1.07)	3.77 (0.87) – 3.56 (1.09)↓†	2.37 (0.49) – 2.29 (0.68)↓†	2.78 (0.86) – 2.61 (0.87)↓†	10.4 (4.3) – 10.4 (4.4)	262.4 (42.8) – 250.8 (39.7)
	Control – Insole B	1.18 (0.10) – 1.18 (0.09)	14.29 (1.51) – 14.34 (1.43)	3.54 (1.06) – 3.31 (1.17)↓†	2.25 (0.77) – 2.12 (0.81)↓†	2.59 (0.85) – 2.41 (0.91)↓†	9.7 (3.7) – 9.0 (3.9)	225.4 (57.9) – 207.7 (56.9)↓†
	Control – Insole C	1.18 (0.10) – 1.17 (0.92)	14.19 (1.84) – 14.46 (1.93)	3.43 (1.32) – 3.60 (1.62)↑†	2.12 (0.75) – 2.37 (1.15)↑†	2.56 (1.15) – 2.56 (1.20)	10.6 (3.8) – 10.8 (3.8)	239.7 (78.5) – 230.6 (69.9)
	Control – Insole D	1.19 (0.11) – 1.18 (0.09)	14.58 (1.58) −14.51 (1.17)	3.63 (1.00) – 3.52 (1.12)↓†	2.19 (0.63) – 2.16 (0.56)↓	2.77 (0.86) – 2.66 (1.03)↓†	9.6 (3.8) – 10.4 (4.5)↑†	230.7 (57.9) – 240.9 (58.0)
Insole less comfortable	Control – Insole A	1.21 (0.05) – 1.16 (0.05)↓†	15.01 (1.33) – 14.21 (1.19)	3.78 (1.18) – 3.38 (1.17)↓†	2.29 (0.87) – 2.22 (0.82)	2.90 (0.85) – 2.44 (0.86)↓*†	9.7 (3.3) – 10.0 (3.4)	246.3 (68.9) – 227.4 (51.4)↓†
	Control – Insole B	1.21 (0.06) – 1.17 (0.06)	15.37 (1.11) – 14.73 (1.01)	3.98 (0.87) – 3.60 (0.78)↓†	2.33 (0.72) – 2.42 (0.59)↑†	3.11 (0.64) – 2.54 (0.56)↓*†	9.7 (3.2) – 10.2 (4.0)	248.1 (52.5) – 224.7 (51.3)↓†
	Control – Insole C	1.19 (0.10) – 1.21 (0.08)	15.11 (1.27) – 15.10 (0.98)	3.76 (0.59) – 3.82 (0.63)	2.24 (0.53) – 2.43 (0.57)↑†	2.91 (0.37) – 2.83 (0.42)↓†	10.4 (3.3) – 11.1 (3.4)	257.2 (49.1) – 268.2 (36.4)
	Control – Insole D	1.82 (0.10) – 1.17 (0.06)	14.89 (1.50) – 14.88 (1.47)	3.76 (0.39) – 3.65 (0.58)↓†	2.38 (0.52) – 2.35 (0.62)	2.80 (0.23) – 2.65 (0.30)↓†	9.5 (3.6) – 9.3 (2.8)	282.3 (75.9) – 255.6 (68.5)↓†

*Note:* Data comparisons presented as: Control Mean (Control SD) – Insole X Mean (Insole X SD). Grey denotes control significantly or meaningfully different to insole; ↑ Test condition significantly or meaningfully greater than control; ↓ Test condition significantly or meaningfully lower than control; * Significant difference (*p* ≤ 0.0125) between insole condition and control; † Meaningful difference between insole condition and control.

There were no significant differences in biomechanical outcome variables when insole conditions were ‘more comfortable’ than the control condition. When insole conditions were ‘less comfortable’ there were significant reductions in braking shock when wearing the test insoles compared with the control condition (insole A and B, Table 5).

When participants reported ‘more comfort’, a meaningful reduction in total shock was reported for three out of the four insole conditions. Impact shock and braking shock were also meaningfully reduced in two out of four, and three out of four conditions respectively. Meaningful decreases in pronation excursion, meaningful increases in total shock, impact shock, and pronation velocity were also observed in single instances of control–insole comparisons.

When participants reported ‘less comfort’ meaningful reductions in biomechanical outcomes were observed in the following number of insole conditions: GRF (1/4), total shock (3/4), braking shock (4/4), and pronation velocity (3/4). Impact shock was meaningfully increased in two out of four insole conditions compared with the control when ‘less comfort’ was reported.

There were only two instances where significant differences between the insole conditions were meaningful as defined by the MCID. In the ‘less comfort’ classification braking shock reduced.

## 4 Discussion

This study is the first to identify the differences in biomechanics that are apparent when individuals report meaningful changes in comfort when wearing different insole conditions in their shoes over a one‐day period. The data collection method offers a protocol for comfort and biomechanical evaluation of orthotic and footwear interventions in clinical settings to increase external validity for assessment. This protocol allows assessment of intervention effectiveness across relevant time periods and environments, as well as the potential to quantify patients wear patterns using a simple combination of a comfort scale and IMU. Grouping participants using a predefined MCID for the 100 mm VAS [20] enabled the study to identify biomechanical differences when adding an insole to a shoe made meaningful changes to comfort. Findings can be applied to the design of footwear‐based interventions in clinical contexts to meaningfully improve comfort whilst maintaining clinical efficacy.

### 4.1 Meaningful Changes in Comfort

Averaged across insole conditions, 12 of the 30 participants rated insoles as meaningfully ‘more comfortable’ than the shoe alone. Whereas nine rated insoles as meaningfully ‘less comfortable’ than the shoe alone. The utility of insoles to meaningfully enhance perceived comfort could be debated based upon these results. Previous research assessing comfort based upon group averages of VAS reports show insoles to improve comfort of a shoe [21, 32]. The current research however demonstrates not all insoles make meaningful differences in footwear comfort for all individuals. Furthermore, when differences are meaningful, they are not always improving comfort and some insoles decreased comfort. Assessing impact of insoles on perceived comfort at the individual level is important as each patient experiences comfort differently, possibly related to anthropometrics [8, 33], pathologies and health conditions [7, 34, 35], and perceptions of different footwear features [8]. This is important for clinicians considering adherence to insole and orthotic interventions when patients may not consider the intervention to be comfortable.

### 4.2 Lower Limb Biomechanics

When meaningfully ‘less comfort’ was reported, there were only two instances where biomechanical outcomes differed significantly between control and insole. Both included significant reductions in braking shock. Statistical testing therefore provided inconclusive evidence that when insoles were ‘less comfortable’ there were any underpinning biomechanical changes. This could be related to limited sample size due to the breakdown of the participant cohort into the comfort classifications, the low *p*‐value required to identify significant differences due to corrections for multiple comparisons, and limitations surrounding design scope for commercial insoles to create large changes in biomechanics.

Considering meaningful change in biomechanics variables; when ‘more’ comfort was reported impact shock commonly meaningfully decreased, and increased when ‘less’ comfort was reported. However, there was one instance where impact shock meaningfully increased in the ‘more’ comfort classification. Impact shock could be a factor by which wearers base comfort perceptions. Other biomechanical variables showed meaningful differences consistent in direction irrespective of whether comfort was ‘more’ or ‘less’. Pronation velocity decreased meaningfully for insoles rated as both meaningfully ‘more’ and ‘less comfortable’. This contrasts the hypothesis that pronation velocity would increase when comfort was reported as meaningfully less. In a clinical situation, we cannot rely on an individual reporting ‘more’ or ‘less’ comfort to imply underlying biomechanical change and intervention effectiveness.

We hypothesised that, in individuals with a comfort classification of ‘less comfort’ in the insole condition, biomechanical parameters would be less favourable (e.g., higher shock, greater pronation range of motion) than in the control. However, non‐significant reductions in biomechanical parameters resulting from insole usage were still observed when individuals reported ‘less comfort’. This included reductions in GRF rate, total shock, and braking shock. The results highlight that comfort perception may not be based upon these parameters, with perception potentially being based on more subjective factors [8, 36].

Results indicate impact shock to be the primary variable underpinning comfort perceptions made by asymptomatic wearers walking in their day‐to‐day lives. Peak positive acceleration and impact shock have been consistently associated with perceptions of footwear comfort [10, 14, 16]. The magnitude of meaningful difference is however very small within the current study, requiring a difference of ± 0.08 g between insoles to be defined as meaningful. This highlights the potential for individuals to sense small differences in impact mechanics resulting from changes in underfoot material properties [37, 38]. Peak tibial acceleration or impact shock can be influenced by thicker insoles made of softer materials [39]. Current results and previous research highlight impact shock to be related to the comfort perception of the wearer. With the relationship between comfort and adherence in clinical settings, clinicians should consider the addition of insole features to absorb shock to improve comfort, potentially driving treatment adherence and patient satisfaction.

Other biomechanical variables showed no significant or meaningful differences in either comfort classification (GRF Rate), or showed the same directional significant and meaningful differences for each comfort classification (Braking shock). Therefore, when participants reported both ‘more’ and ‘less’ comfort, changes in biomechanics from the control to insole condition were equivalent. This may highlight that comfort is dependent on sensations that cannot be categorised by biomechanics, or specifically the RunScribe IMU. In qualitative studies investigating footwear comfort, participants describe comfortable footwear as being strongly related to cushioning [36]. Previous biomechanical analyses have linked greater cushioning properties (thicker shoe midsoles), to reduced loading rates during running [40, 41]. It could therefore be assumed that ground reaction forces play a role within perceived footwear comfort. However, a clear pattern of meaningful or statistical changes in ground reaction force variables with meaningful changes in comfort was not evident in this study. Clinicians prescribing footwear or insole‐based interventions should consider the likely perceptions of the wearer alongside the required differences in biomechanics in relation to the pathology. This would enable development of footwear‐based interventions that improve comfort to both drive adherence and maintain their clinical efficacy for treatment.

### 4.3 Limitations

The pre‐defined MCID [20] was key to the interpretation of this research, but defined on shoes during running, whereas the current study examined insoles during daily living activity (predominantly walking). The language of the anchors on the VAS was also different, with ‘least comfort imaginable’ and ‘most comfort imaginable’ within the current study, compared with ‘not comfortable at all’ and ‘most comfort imaginable’ (20). This method was however chosen as the only defined MCID available within the literature for the 100 mm VAS. The study questions the ability for insoles to create meaningful changes in lower limb biomechanics, evidenced by limited differences presented in Table 2. The limited design scope available for insoles within a commercial context causes difficulty creating large biomechanical differences, reflected in the limited number of meaningful differences within the study. The lack of knowledge of exact design specification of the insoles limited the interpretation of insole effect. Without this information, a definite hypothesis of the effectiveness of each insole could not be presented.

The population group was an asymptomatic convenience sample; therefore, the results of the current study may not reflect the outcomes within a clinical group. To understand how these outcomes relate to different clinical populations or symptomatic groups, further exploration would be required. Translation of the method to a clinical group could involve assessment of custom versus off‐the‐shelf devices, or assessment of a range of footwear‐based interventions to identify the most effective treatment method based on meaningful changes and lower limb biomechanics measured over longer periods of time in real‐world settings.

## 5 Conclusion

This is the first study to assess wearers self‐reported insole comfort and changes in biomechanics over one day of wear. The RunScribe IMU served as a portable method of biomechanics data collection allowing clinicians and practitioners to assess changes in biomechanics following footwear and orthotic intervention as treatment, or for collection of clinical patient group assessment related to pathologies. Results show that reductions in impact shock are evident when comfort meaningfully increases, as are increases in impact shock when comfort meaningfully decreases. This was the only consistent contrast between the ‘more’ and ‘less’ comfort groups for the differences in biomechanics between insole conditions. Within a clinical context, the current study provides interpretation of biomechanics data alongside meaningful changes in comfort of insole products. This enables a greater understanding of how footwear comfort during a day of wear can be impacted by changes in biomechanical parameters within real‐life activities. Decisions can also be made around severity of footwear interventions to allow for suitable biomechanics changes without compromising wear comfort driving adherence to footwear‐based treatments.

## Author Contributions

**Max Lewin:** conceptualization, data curation, formal analysis, investigation, methodology, project administration, writing – original draft, writing review and editing. **Richard K. Jones:** conceptualization, supervision, writing – review and editing. **Carina L. Price:** conceptualization, funding acquisition, methodology, project administration, supervision, writing – original draft, writing – review and editing.

## Funding

This study comes from a project funded by Innovate UK (ukri.org/councils/innovate‐uk/) and Scholl's Wellness Company (scholl.co.uk), project number KTP011560. The funders had no role in study design, data collection and analysis, decision to publish, or preparation of the manuscript.

## Ethics Statement

Favourable opinion was given by the University of Salford School of Health and Society ethics panel (# 6395).

## Consent

Written consent was obtained from all participants via consent form prior to any participation within the study protocol.

## Conflicts of Interest

The authors declare no conflicts of interest.

## Supporting information


Supporting Information S1

## Data Availability

The data that support the findings of this study are available on request from the corresponding author. The data are not publicly available due to privacy or ethical restrictions.
