# Supplementary material for: Do Commercially Available Insoles Meaningfully Change Reported Comfort and Biomechanics During One Day of Wear?
Source: J Foot Ankle Res. 2026 May 14;19(2):e70163. doi: 10.1002/jfa2.70163 (PMC13175921; doi:10.1002/jfa2.70163)
Supplement: Supplementary file 1 — Supporting Information S1 [file JFA2-19-e70163-s001.docx]

A (3,4) Intraclass correlation analysis with average measures was completed using SPSS statistics 29 (IBM, Armonk, New York, USA) to assess the repeatability of the RunScribe over multiple days of measurement.

Method: Ten participants completed 4 days of repeated measurements using their own footwear whilst completing their normal daily activities. The definition of the normal daily activity did not differ to that used within the larger study. Participants were requested to complete the four days of measurement on days which they would complete the same daily activities to ensure the walking data collected would be as repeatable as possible.

The RunScribe unit was applied and calibrated on each day of measurement and removed at the end of the day. Removing the potential impact of different placements of the RunScribe measurement unit on each repeated day of measurement. During the wear period the RunScribe measured vertical GRF, vertical GRF rate, Pronation excursion, maximum pronation velocity, impact shock, braking shock, and total shock.

Results: The ICC showed excellent levels of agreement between the repeated measures of peak GRF (0.915), pronation excursion (0.983), pronation velocity (0.982), impact shock (0.984), braking shock (0.987), and total shock (0.987). There was however only moderate agreement for repeated measurements of GRF rate (0.673).
